# Association Analysis of Neuronal Nitric Oxide Synthase 1 Gene Polymorphism With Psychopathological Symptoms in Chronic Ketamine Users

**DOI:** 10.3389/fpsyt.2020.580771

**Published:** 2020-12-23

**Authors:** Jiansong Chen, Minling Zhang, Chao Zhou, Yi Ding, Ni Fan, Hongbo He

**Affiliations:** The Affiliated Brain Hospital of Guangzhou Medical University, School of Mental Health, Guangzhou Medical University, Guangzhou, China

**Keywords:** nitric oxide synthase 1, single nucleotide polymorphisms, ketamine use, association analysis, psychopathological symptoms

## Abstract

**Objective:** We previously found that chronic ketamine usages were associated with various psychotic and cognitive symptoms mimicking schizophrenia. The blockade of the NMDA receptor and subsequent nitric oxide synthase 1 (NOS1) dysfunction were found to be closely correlated with schizophrenia including NOS1 gene polymorphisms. We examined the allelic variants of the gene coding neuronal nitric oxide synthase 1 (*NOS1*) in chronic ketamine users in the Chinese population and analyzed the association between *NOS1* gene polymorphism and psychopathological symptoms in chronic ketamine users. The association between the *NOS1* polymorphism and ketamine use characteristics was also examined.

**Methods:** One hundred ninety seven male chronic ketamine users and 82 controls were recruited. Four common SNPs of the *NOS1* gene, rs6490121, rs41279104, rs3782206, and rs3782219, were examined by real-time PCR with the TaqMan® assay system. Psychopathological symptoms were assessed using the Positive and Negative Syndrome Scale (PANSS), Beck Depression Inventory (BDI), and the Beck Anxiety Inventory (BAI).

**Results:** The genotype distribution of rs6490121 and rs41279104 in chronic ketamine users was significantly different from that in the control (*p* = 0.0001 and *p* = 0.002). The G allele frequency of rs6490121 in ketamine users was higher than that in the control (*p* = 2.23 * 10^−6^, OR = 3.07, 95% CI = 1.93–4.90). The T allele frequency of rs41279104 in chronic ketamine users was higher than that in the control (*p* = 0.01, OR = 1.76, 95% CI = 1.14–2.72). The BAI score was significantly different among the three genotypic groups of rs6490121 (*F* = 6.21, *p* = 0.002) in ketamine users; subjects of genotype AG and GG had a lower score than subjects of genotype AA. The score of the negative symptom subscale of PANSS was significantly different among the three genotypic groups of rs41279104 (*F* = 5.39, *p* = 0.005); in ketamine users, subjects of genotype CT and TT had a higher score than subjects of genotype CC. There was no difference in drug use characteristics in different genotypes of the four *NOS1* gene polymorphisms tested in ketamine users (*p* > 0.05).

## Introduction

Ketamine, a derivative of phencyclidine (PCP), is an N-methyl-D-aspartic acid receptor (NMDAR) antagonist; NMDAR was widely associated with many critical physiological processes like neurodevelopment, neuroplasticity, neurocognition, sensory, and mood function (1, 2). Ketamine has been widely abused as a psychoactive substance since the late 1990s (3), and it is the third most commonly abused substance after morphine and methamphetamine in China (4).

Acute administration of ketamine in healthy volunteers induces schizophrenic-like symptoms including positive and negative symptoms and cognitive impairment (5–8). Our study showed that chronic ketamine users exhibited greater similarity than the acute ketamine injection in the normal control group to the symptom domains of schizophrenia (9). Ketamine administration was considered as a pharmacological model in studying schizophrenia, and chronic ketamine users could potentially be a natural human model for schizophrenic research especially in exploring the various physiological consequences after chronic NMDAR blockade in the human brain (10–13).

Alteration of the nitric oxide (NO) level via NMDAR blockade and subsequent nitric oxide synthase 1 (NOS1) dysfunction was found to be closely correlated with schizophrenia (14). NMDAR, NOS1, and postsynaptic density protein 95 (PSD95) form a tertiary protein complex (15). Phencyclidine (PCP) was another NMDAR blocker and was widely used in schizophrenia animal models. Blockade of NOS1 was found to enhance PCP-induced schizophrenia-like symptoms and to increase c-fos gene expression in the cortical region (16). Moreover, PCP cannot induce psychotic symptoms in NOS1 knockout mice (17). On the contrary, the NO donor can block these changes and effectively eliminate the psychotic symptoms induced by NMDAR blockers and improve cognitive function (16, 18, 19). Clinical studies found that levels of NO metabolites were lower in plasma and cerebrospinal fluid of patients with schizophrenia than that in normal individuals and increased after 8 weeks of risperidone treatment and the NO metabolite concentration was negatively correlated with the severity of negative symptoms in patients (14, 20). The genetic polymorphisms of *NOS1* of the NMDAR-NO-cGMP pathway were also found to be closely related to schizophrenia (21, 22). *NOS1* is located in 12q24.2 and contains a total of 29 exons with a total length of ~149.4 kb (https://www.genecards.org/). *NOS1* is a relevant functional and positional candidate gene for schizophrenia (23).

Clinically, it has been observed that only some ketamine users developed ketamine-related psychosis while others might not develop psychotic symptoms even if they used higher doses and longer durations (24). Moreover, some psychotic symptoms developed after ketamine use could last for weeks to months or even longer after stopping ketamine usage (25). Since the ketamine model is one of the most common schizophrenic models, there is no study to test whether the genetic variation of *NOS1* is involved in the occurrence of psychosis in some chronic ketamine users. Based on previous evidences, we hypothesized that SNPs of *NOS1* may play a role in ketamine-induced psychosis.

The present study tested four SNPs of *NOS1* (rs6490121, rs41279104, rs3782206, rs3782219) commonly found in schizophrenic patients to determine if there were differences in genotypes and allele distributions of *NOS1* gene polymorphism between ketamine users and healthy controls. We also analyzed the association between *NOS1* gene polymorphism and the severity of psychopathological symptoms and the relationship between *NOS1* gene polymorphism and ketamine use characteristics in ketamine users.

## Materials and Methods

### Subject Recruitment

A total of 197 chronic ketamine users and 82 controls were recruited. All ketamine users and controls were male. Chronic ketamine users were recruited voluntarily at Guangzhou Baiyun Voluntary Medical Detoxification Hospital. Healthy controls were recruited through advertisement. All chronic ketamine users underwent a 2-h semistructured interview which was conducted by clinicians with 3 or more years of clinical experience. Inclusion criteria required (1) chronic ketamine users be admitted for detoxification or treatment of ketamine-related symptoms; (2) no other substance dependence other than ketamine and tobacco according to DSM-IV-TR; and (3) subjects use ketamine as a drug of choice for longer than 6 months. Ketamine subjects with a history of psychiatric and neurological diseases prior to ketamine use disorder were excluded. The control subjects were free from a history of drug abuse or psychiatric disorder. Written informed consent for this study was provided by all subjects, and the study was approved by the Institutional Ethics Committee.

### Assessment of Psychotic Symptoms

Clinical symptoms among ketamine users were evaluated with the Positive and Negative Syndrome Scale (PANSS) (26), administered by two trained psychiatrists with 3 or more years of clinical experience. The intraclass correlation coefficient (ICC) between raters on the PANSS was >0.8. In addition, ketamine users were asked to complete the Beck Depression Inventory (BDI) (27) and the Beck Anxiety Inventory (BAI) (28), which assessed their depressive and anxiety symptoms during the 2 weeks before they were hospitalized.

### Sample Collection and Genotyping

Briefly, peripheral blood samples were collected from subjects in 6-ml EDTA vacuum tubes. Genomic DNA was extracted from peripheral blood using the standard phenol–chloroform extraction method (29). The final concentrations of all DNA samples were 20 ng/μl with good purity (OD260/OD280 = 1.8–2.0) and stored at −80°C until genotyping. The TaqMan real-time quantitative PCR (q-PCR) method was used to test DNA samples using the TaqMan® Genotyping Assay (cat#25800731, cat#27481263, cat#86363451, cat#29122242, Applied Biosystems, Shanghai, China) on an ABI ViiA™ 7 sequence detection system instrument (Applied Biosystems). PCR was performed in 25-μL reactions containing Genotyping Master Mix (Applied Biosystems, Shanghai, China), TaqMan® Genotyping Assay, and DNA template. Each 96-well-plate was quality-controlled with two no-template controls. The PCR program for the amplification was as follows: 95°C for 10 min and then 40 cycles of denaturation (95°C for 15 s) and annealing (60°C for 1 min). We further randomly selected 10% samples for each of the selected SNPs for re-genotyping, and the results were 100% concordant.

### Statistical Analysis

Demographics and clinical data of ketamine users were statistically analyzed with μ ± SD. The Hardy–Weinberg equilibrium of all pairs of SNPs was calculated using SHEsis software (http://analysis.bio-x.cn) (30). Chi-square tests were used to compare the allelic and genotypic frequencies between ketamine users and healthy controls. Odds ratios (ORs) were used to measure the association of genotypes of the four SNPs between ketamine users and control subjects. Logistic regression analysis was used to obtain ORs and their 95% confidence intervals (95% CI). Age and years of education were evaluated as confounding factors. Bonferroni corrections were applied to adjust for multiple comparisons of different genotypes of SNPs in ketamine users and control subjects. To adjust for multiple comparisons, the significance level was set to *p* < 0.017 (α/3 = 0.05/3, two-tailed) for all multiple comparisons. Between-group differences grouped by genotypes were evaluated using one-way analysis of variance (ANOVA), followed by Fisher's least significant difference (LSD) multiple-range test for between-group comparison. Statistical analyses were performed using the Statistical Package for the Social Sciences (SPSS) version 22.0. The level of significance was set at α = 0.05 (except multiple comparisons).

## Results

### Participant Characteristics

Socio-demographic characteristics, drug use conditions, and PANSS, BDI, and BAI scores are presented in Table 1. Age and years of education were significantly different between the two groups (*p* < 0.001).

**Table 1:** Demographics and clinical data of ketamine users and controls^*^.

	**Ketamine users (*n* = 197)**	**Controls (*n* = 82)**
Age (years)	25.83 ± 4.84	33.61 ± 10.97^**^
Education (years)	10.47 ± 2.67	12.21 ± 2.66^**^
Age of first ketamine use, years ± SD	19.44 ± 4.65	–
Duration of ketamine use, years ± SD	6.54 ± 2.98	–
Average daily dose, g	2.97 ± 2.42	–
Frequency of ketamine use weekly	6.14 ± 1.67	–
PANSS score	44.89 ± 8.28	–
Positive symptom subscale	7.97 ± 1.82	–
Negative symptom subscale	12.95 ± 3.61	–
General psychopathology subscale	23.96 ± 4.93	–
BDI score	13.09 ± 6.39	–
BAI score	18.89 ± 12.57	–

* *All subjects were male*.

** *p < 0.001*.

### Genotype and Allele Frequencies of SNPs in Ketamine Users and Control Subjects

Genotypes and allele frequencies of the four SNPs in gene *NOS1* are shown in Tables 2, 3. Distributions of each SNP genotype were consistent with Hardy–Weinberg equilibrium (HWE) in healthy controls (*p* > 0.05). The genotype distribution of rs6490121 and rs41279104 had a significant difference between ketamine users and control subjects (*p* = 0.0001 and *p* = 0.002), respectively. Genotypes GG and TT in ketamine users were higher than those in normal control. The G allele frequency of rs6490121 in ketamine users was higher than that in the controls (*p* = 2.23 * 10^−6^, OR = 3.07, 95% CI = 1.93–4.90). The T allele frequency of rs41279104 in ketamine users was higher than that in the controls (*p* = 0.01, OR = 1.76, 95% CI = 1.14–2.72). For the other two SNPs (rs3782206 and rs3782219), neither the genotype distributions nor allele frequencies were significantly different between ketamine users and control subjects (*p* > 0.05).

**Table 2:** Distribution of genotypes of *NOS1* gene in ketamine users vs. control subjects.

**SNP ID**	**Subjects**	***N***	**HWE-p**	**Genotype** ***n*** **(%)**			**Adjusted χ2**	***p***
				**AA**	**AG**	**GG**		
rs6490121 (A>G)	KET	197	0.272	95 (48.2)	68 (34.5)	34 (17.3)	18.13	0.0001^a^^*^
	CON	82		61 (74.4)	18 (22.0)	3 (3.6)		
				**CC**	**CT**	**TT**		
rs41279104 (C>T)	KET	197	0.826	114 (57.9)	45 (22.8)	38 (19.3)	12.21	0.002^b^^*^
	CON	82		52 (63.4)	27 (32.9)	3 (3.7)		
				**CC**	**CT**	**TT**		
rs3782206 (C>T)	KET	197	0.803	125 (63.5)	54 (27.4)	18 (9.1)	4.01	0.13
	CON	82		48 (58.5)	30 (36.6)	4 (4.9)		
				**CC**	**CT**	**TT**		
rs3782219 (C>T)	KET	197	0.991	82 (41.6)	85 (43.1)	30 (15.3)	0.29	0.86
	CON	82		33 (40.2)	38 (46.3)	11 (13.5)		

a *GG compared with AA and AG*.

b *TT compared with CC and CT*.

* *p < α/3 = 0.05/3*.

**Table 3:** Allele frequency of the *NOS1* gene in ketamine users vs. control subjects.

**SNP ID**	**Subjects**	**Allele** ***N*** **(%)**		**Adjusted χ2**	***p***	**Adjusted OR (95% CI)**
		**G**	**A**			
rs6490121 (A>G)	KET	136 (34.5)	258 (65.5)	22.39	2.23*10^**−6**^	**3.07 (1.93–4.90)**
	CON	24 (14.6)	140 (85.4)			1.00 (ref.)
		**T**	**C**			
rs41279104 (C>T)	KET	121 (30.7)	273 (69.3)	6.50	**0.01**	**1.76 (1.14–2.72)**
	CON	33 (20.1)	131 (79.9)			1.00 (ref.)
		**T**	**C**			
rs3782206 (C>T)	KET	90 (22.8)	304 (77.2)	0.007	0.93	0.98 (0.64–1.51)
	CON	38 (23.2)	126 (76.8)			1.00 (ref.)
		**T**	**C**			
rs3782219 (C>T)	KET	145 (36.8)	249 (63.2)	0.002	0.96	1.01 (0.69–1.47)
	CON	60 (36.6)	104 (63.4)			1.00 (ref.)

### Relationship Between Psychotic Symptom Measures and Genotypes of Four NOS1 SNPs

Associations between psychotic symptom measures and genotypes of four SNPs in ketamine users are shown in Table 4. The BAI score was significantly different among the three genotypic groups of rs6490121 (*F* = 6.21, *p* = 0.002) in ketamine users; subjects of genotype AG and GG had a lower score than subjects of genotype AA (respectively, *p* = 0.043, *p* = 0.037). The score of the negative symptom subscale was significantly different among the three genotypic groups of rs41279104 (*F* = 5.39, *p* = 0.005); subjects of genotype CT and TT had a higher score than subjects of genotype CC (respectively, *p* = 0.048, *p* = 0.045). There were no significant genotype effects on rs3782206 and rs3782219 (all *p* > 0.05).

**Table 4:** Association between psychotic symptom measures and genotypes of four *NOS1* SNPs in ketamine users.

	**Genotype**			***F***	***p***
rs6490121	**AA (95)**	**AG (68)**	**GG (34)**		
PANSS total score	44.16 ± 7.66	45.24 ± 9.29	46.21 ± 7.80	0.86	0.42
Positive symptom subscale	7.89 ± 2.02	8.04 ± 1.72	8.03 ± 1.45	0.16	0.85
Negative symptom subscale	12.64 ± 3.40	12.96 ± 3.77	13.82 ± 3.78	1.35	0.26
General psychopathology subscale	23.63 ± 4.45	24.24 ± 5.84	24.35 ± 4.26	0.43	0.65
BDI score	13.0 ± 6.27	13.15 ± 6.37	13.21 ± 6.92	0.02	0.98
BAI score	22.07 ± 14.47	16.09 ± 9.87^a^	15.59 ± 9.40^b^	**6.21**	**0.002**
rs41279104	**CC(114)**	**CT(45)**	**TT(38)**		
PANSS total score	43.96 ± 8.45	45.44 ± 8.52	46.97 ± 7.18	2.03	0.13
Positive symptom subscale	7.82 ± 1.66	8.42 ± 2.46	7.87 ± 1.28	1.83	0.16
Negative symptom subscale	12.67 ± 3.76	12.27 ± 2.97^c^	14.61 ± 3.42^d^	**5.39**	**0.005**
General psychopathology subscale	23.47 ± 5.03	24.76 ± 5.16	24.5 ± 4.29	1.38	0.25
BDI score	12.65 ± 6.66	12.86 ± 6.2	14.63 ± 5.63	1.41	0.25
BAI score	19.87 ± 13.88	17.95 ± 11.47	17.05 ± 9.19	0.87	0.42
rs3782206	**CC(125)**	**CT(54)**	**TT(18)**		
PANSS total score	44.67 ± 8.03	45.11 ± 8.95	45.72 ± 8.27	0.15	0.86
Positive symptom subscale	7.85 ± 1.50	8.28 ± 2.43	7.89 ± 1.75	1.07	0.35
Negative symptom subscale	12.74 ± 3.58	13.09 ± 3.60	14.0 ± 3.82	1.02	0.36
General psychopathology subscale	24.08 ± 4.91	23.74 ± 4.87	23.83 ± 5.53	0.10	0.91
BDI score	12.94 ± 6.45	13.20 ± 6.28	13.72 ± 6.56	0.13	0.88
BAI score	20.54 ± 13.38	16.13 ± 10.67	15.83 ± 10.48	2.97	0.06
rs37822019	**CC(82)**	**CT(85)**	**TT(30)**		
PANSS total score	45.53 ± 8.65	44.28 ± 8.51	44.87 ± 6.53	0.47	0.62
Positive symptom subscale	8.09 ± 1.93	7.85 ± 1.84	8.0 ± 1.49	0.36	0.70
Negative symptom subscale	13.27 ± 3.52	12.64 ± 3.66	13.0 ± 3.74	0.64	0.53
General psychopathology subscale	24.17 ± 5.32	23.80 ± 5.08	23.87 ± 3.27	0.12	0.88
BDI score	13.84 ± 6.36	12.53 ± 6.06	12.67 ± 7.34	0.96	0.39
BAI score	19.33 ± 12.5	18.91 ± 13.10	17.67 ± 11.49	0.19	0.83

a *p = 0.043, compared with AA genotype, LSD post-hoc test*.

b *p = 0.037, compared with AA genotype, LSD post-hoc test*.

c *p = 0.048, compared with CC genotype, LSD post-hoc test*.

d *p = 0.045, compared with CC genotype, LSD post-hoc test*.

### Relationship Between Drug Use Characteristics and Genotypes of Four NOS1 SNPs

The association between drug use characteristics and genotypes of the four *NOS1* SNPs in ketamine users was analyzed (Table 5). No significant differences in drug use characteristics among genotype groups of rs6490121, rs41279104, rs3782206, and rs3782219 were observed (*p* > 0.05).

**Table 5:** Association between drug use characteristics and genotypes of four *NOS1* SNPs in ketamine users.

	**Genotype**			***F***	***p***
rs6490121	**AA (95)**	**AG (68)**	**GG (34)**		
Age of first ketamine use	19.97 ± 4.66	18.97 ± 4.36	18.9 ± 5.17	1.19	0.31
Duration of ketamine use	6.13 ± 2.78	6.82 ± 2.85	7.12 ± 3.68	1.85	0.16
Average daily dose	3.0 ± 2.45	2.97 ± 2.53	2.89 ± 2.15	0.03	0.97
Frequency of use weekly	6.34 ± 1.49	6.01 ± 1.77	5.85 ± 1.93	1.40	0.25
rs41279104	**CC (114)**	**CT (45)**	**TT (38)**		
Age of first ketamine use	19.16 ± 4.46	20.0 ± 4.79	19.63 ± 5.11	0.56	0.57
Duration of ketamine use	6.65 ± 3.24	6.29 ± 2.54	6.49 ± 2.73	0.24	0.79
Average daily dose	2.92 ± 2.39	3.08 ± 2.32	3.03 ± 2.66	0.08	0.92
Frequency of use weekly	6.35 ± 1.53	5.86 ± 1.84	5.86 ± 1.83	2.16	0.12
rs3782206	**CC (125)**	**CT (54)**	**TT (18)**		
Age of first ketamine use	19.52 ± 5.11	19.43 ± 3.86	18.9 ± 3.51	0.14	0.87
Duration of ketamine use	6.76 ± 3.32	6.28 ± 2.20	5.76 ± 2.53	1.16	0.32
Average daily dose	2.97 ± 2.36	3.13 ± 2.72	2.54 ± 1.87	0.40	0.67
Frequency of use weekly	6.12 ± 1.70	6.3 ± 1.54	5.83 ± 1.93	0.56	0.57
rs3782219	**CC (82)**	**CT (85)**	**TT (30)**		
Age of first ketamine use	19.41 ± 4.36	19.33 ± 4.89	19.83 ± 4.9	0.13	0.88
Duration of ketamine use	6.27 ± 2.65	6.78 ± 3.29	6.58 ± 2.98	0.61	0.54
Average daily dose	3.38 ± 2.57	2.7 ± 2.2	2.61 ± 2.45	1.41	0.25
Frequency of use weekly	6.34 ± 1.52	6.04 ± 1.75	5.87 ± 1.85	1.13	0.33

## Discussion

To our knowledge, this is the first study to investigate the gene polymorphism of *NOS1* in chronic ketamine users. We found more genotypic variations of rs6490121 and rs41279104 in ketamine users than those in normal control. Among ketamine users, the BAI score was significantly lower with the genotypic variation of rs6490121 and the score of the PANSS negative symptom subscale was significantly higher in the genotypic TT group of rs41279104. There is no significant correlation between tested gene polymorphism with ketamine use characteristics.

In previous schizophrenic studies, it has been found that variations in the locus of the functional promoter region of SNP rs41279104 leads to a decrease in *NOS1* gene expression and the variation of rs41279104 results in decreased prefrontal lobe expression of *NOS1*, which consequently reduced brain executive function, increased error in continuous operation testing, and increased P300 latency (31). In the high-density EEG study, the rs41279104 G allele carrier showed a decrease in P1 response compared to non-carriers (32). Another SNP rs6490121 of *NOS1* was also found to be associated with working memory impairment in schizophrenic patients, and the working memory impairment of risk allele carriers was declining (33).

Our results were in line with these findings in schizophrenia studies. The rs41279104 and rs6490121 variations were identified in chronic ketamine users similar to that found in schizophrenia. Moreover, such variations were found closely correlated with lower anxiety symptoms and higher negative symptoms not associated with positive symptoms. Although the cognitive assessment was not included in our current analysis, the PANSS negative symptoms also include part of cognition test items and the score of negative symptoms was found to be highly related with the cognitive functions (34, 35). Our previous study analyzed the short-term and long-term symptom features in 187 chronic ketamine users and found that the short-term symptoms were closer to the positive symptoms, while long-term symptoms were more similar to the schizophrenic negative symptoms including more social withdrawal symptoms like personality change, being unsociable and eccentric, reduced social activities, reduced number of friends, and cognitive symptoms including memory loss, slow reaction time, and decreased work efficiency (36). It suggested that such variations were potentially more related with cognitive impairment and negative symptoms instead of positive psychotic symptoms as shown in schizophrenia. The presence of critical genetic variations in the *NOS1* gene in ketamine users could increase the susceptibility of schizophrenic negative symptoms and potentially with more cognitive impairment while being challenged by chronic ketamine usage which could further reduce the functioning of NOS1 by upstream NMDAR blockade. Further studies are needed to investigate the role of NOS1 and NO in ketamine-related psychosis.

The present study did not detect significant differences of the other two *NOS1* SNPs (rs3782206 and rs3782219). However, a study reported that there is a link at rs3782206 and cognitive functions, which underpinned at the inferior frontal gyrus (23). Another study showed that a link between *NOS1* gene polymorphism at rs3782206 and cognitive functions and their neural underpinnings at the IFG (22). The absence of a positive finding in our study suggests that any effect of these gene polymorphisms on ketamine users might be weak in the Chinese population.

Although we did not found relationship between *NOS1* polymorphisms and ketamine use characteristics, it has been found that mutation of the *NOS1* gene substantially increased vulnerability to alcohol-induced cell loss in a brain region where the gene is expressed (37). Targeted disruption of the *NOS1* gene worsens the behavioral impact of developmental alcohol exposure and allows alcohol-induced learning problems to emerge (38). Findings of roles for NO and *NOS1* in alcohol-related mental disease indicate that further studies are needed to explore the role of NO and *NOS1* in ketamine users.

Besides *NOS1*, other gene variations were also found to be related with the function of ketamine. A study found that brain-derived neurotrophic factor (BDNF) Val66Met polymorphism was a susceptible gene for ketamine abuse. BDNF Val66Met polymorphism leads to the decrease of BDNF concentration in brain tissue, which resulted in the change of addiction behavior (39). The resting-state functional connectivity (rs-fc) reduction was more pronounced in norepinephrine transporter (NET) rs28386840 [AA] homozygous subjects than in [T] carriers after ketamine administration, which may contribute to understanding the antidepressant effect of ketamine (40). It implicated that more genetic variations besides the NMDAR-NOS1-NO pathway were correlated with ketamine's effects and more exploratory studies were needed to indentify the critical factors contributing to ketamine addiction and to ketamine-induced psychotic symptoms.

A few limitations of this study have to be mentioned. Firstly, only four SNPs of *NOS1* were examined. Further genetic and mechanism studies with more variants of the *NOS1* gene and other genes would be necessary. Secondly, all ketamine users were male and in-patients, and the sample size was relatively small for such genetic studies. In a further study, subjects from the community with larger samples size should be considered. Thirdly, the control group was not matched in age and education, since ketamine users were mainly male with a relatively low education level and it was hard to recruit a control group with a perfect match. We set age and education as the covariables during group comparisons to minimize its possible confounding effects. Lastly, some ketamine users had used other psychoactive drugs other than ketamine. It may be a confounding factor for the study. However, all the subjects recruited used ketamine as a drug of choice for longer than 6 months and they had no other substance dependence other than ketamine and tobacco. So confounding is likely to be limited.

## Conclusion

Our study showed that chronic ketamine users carried more genotypic variations of rs6490121 and rs41279104 in the *NOS1* gene than the control group and such variations were associated with more negative psychotic symptoms and fewer anxiety symptoms. It implicated that patients with genetic variations in the *NOS1* gene potentially were more vulnerable to chronic ketamine usages, and it warranted further studies to use chronic ketamine users as models to explore the role of NOS1 dysfunction in ketamine-induced psychological symptoms.

## Data Availability Statement

According to national legislation/guidelines, specifically the Administrative Regulations of the People's Republic of China on Human Genetic Resources (http://www.gov.cn/zhengce/content/2019-06/10/content_5398829.htm, http://english.www.gov.cn/policies/latest_releases/2019/06/10/content_281476708945462.htm), no additional raw data is available at this time. Data of this project can be accessed after an approval application to the China National Genebank (CNGB, https://db.cngb.org/cnsa/). Please refer to https://db.cngb.org/, or email: CNGBdb@cngb.org for detailed application guidance. The accession code CNP0001402 should be included in the application.

## Ethics Statement

The studies involving human participants were reviewed and approved by Institutional Ethics Committee of affiliated Brain Hospital of Guangzhou Medical University. The patients/participants provided their written informed consent to participate in this study.

## Author Contributions

HH and NF designed the study, wrote the protocol, and helped on the manuscript revision. YD and CZ recruited the subjects and assessed the psychotic symptoms. MZ collected the blood samples. JC undertook the genotyping and statistical analysis and wrote the manuscript. All authors contributed to and have approved the final manuscript.

## Conflict of Interest

The authors declare that the research was conducted in the absence of any commercial or financial relationships that could be construed as a potential conflict of interest.
